# Biogeography of Photosynthetic Light-Harvesting Genes in Marine Phytoplankton

**DOI:** 10.1371/journal.pone.0004601

**Published:** 2009-02-25

**Authors:** Thomas S. Bibby, Yinan Zhang, Min Chen

**Affiliations:** 1 School of Ocean and Earth Sciences, National Oceanography Centre, Southampton, United Kingdom; 2 School of Biological Sciences, University of Sydney, Sydney, New South Wales, Australia; Mt. Alison University, Canada

## Abstract

**Background:**

Photosynthetic light-harvesting proteins are the mechanism by which energy enters the marine ecosystem. The dominant prokaryotic photoautotrophs are the cyanobacterial genera *Prochlorococcus* and *Synechococcus* that are defined by two distinct light-harvesting systems, chlorophyll-bound protein complexes or phycobilin-bound protein complexes, respectively. Here, we use the Global Ocean Sampling (GOS) Project as a unique and powerful tool to analyze the environmental diversity of photosynthetic light-harvesting genes in relation to available metadata including geographical location and physical and chemical environmental parameters.

**Methods:**

All light-harvesting gene fragments and their metadata were obtained from the GOS database, aligned using ClustalX and classified phylogenetically. Each sequence has a name indicative of its geographic location; subsequent biogeographical analysis was performed by correlating light-harvesting gene budgets for each GOS station with surface chlorophyll concentration.

**Conclusion/Significance:**

Using the GOS data, we have mapped the biogeography of light-harvesting genes in marine cyanobacteria on ocean-basin scales and show that an environmental gradient exists in which chlorophyll concentration is correlated to diversity of light-harvesting systems. Three functionally distinct types of light-harvesting genes are defined: (1) the phycobilisome (PBS) genes of *Synechococcus*; (2) the *pcb* genes of *Prochlorococcus*; and (3) the iron-stress-induced (*isiA*) genes present in some marine *Synechococcus*. At low chlorophyll concentrations, where nutrients are limited, the Pcb-type light-harvesting system shows greater genetic diversity; whereas at high chlorophyll concentrations, where nutrients are abundant, the PBS-type light-harvesting system shows higher genetic diversity. We interpret this as an environmental selection of specific photosynthetic strategy. Importantly, the unique light-harvesting system *isiA* is found in the iron-limited, high-nutrient low-chlorophyll region of the equatorial Pacific. This observation demonstrates the ecological importance of *isiA* genes in enabling marine *Synechococcus* to acclimate to iron limitation and suggests that the presence of this gene can be a natural biomarker for iron limitation in oceanic environments.

## Introduction

In oceanic systems, oxygenic photosynthesis is performed by microbial phytoplankton, the prokaryotic component of which is dominated by two cyanobacterial genera *Synechococcus* spp and *Prochlorococcus* spp [1]–[4], known as oxyphotobacteria. These two genera can coexist [5]; however, *Synechococcus* are the dominant genera at temperate latitudes and coastal regions [3], [4] where nutrient concentrations and biomass are relatively high, and *Prochlorococcus* dominate in tropical latitude ocean gyres [3], [6]–[8] where nutrient concentrations and biomass are relatively low [3].

*Synechococcus* and *Prochlorococcus* are defined by two distinct light-harvesting (LH) systems that act as LH antenna for both types of photosynthetic reaction center, photosystem I (PSI) and photosystem II (PSII) [9], [10]. The LH system in *Synechococcus* involves the phycobilisome (PBS), stacks of chromophorylated protein complexes located externally to the photosynthetic thylakoid membrane and encoded by the genes *cpc* (phycocyanin), *cpe* (phycoerythrin) and *apc* (allophycocyanin) [11]. Some *Prochlorococcus* strains have *cpe* genes; however, these are phylogenetically distinct from *Synechococcus cpe* [12] and no *Prochlorococcus* has been shown to synthesize a functional phycobilisome; indeed, the role of phycoerythrin is thought to be signal transduction rather than light harvesting [13]. The LH system in *Prochlorococcus* involves membrane-bound, chlorophyll-binding proteins (Pcbs) encoded by the *pcb* genes [14]–[18]. Some marine *Synechococcus* contain *pcb*-like genes that are induced under conditions of iron limitation and can be identified as a phylogenetically distinct group that includes the functionally characterized iron-stress-induced gene *isiA*; this gene is sometimes referred to as *pcbD or pcbC/isiA* and here is called *isiA*-like [6], [19]–[21]. We can therefore define three functionally distinct types of LH genes in marine oxyphotobacteria: (1) the PBS genes of *Synechococcus*; (2) the *pcb* genes of *Prochlorococcus*; and (3) the iron-stress-induced genes (*isiA*-like) present in some marine *Synechococcus*.

The Global Ocean Sampling Project (GOS) is revolutionizing our understanding of the complexity of marine microbial communities that drive biogeochemical cycles [22]–[26]. It provides a unique and powerful tool with which the environmental diversity of a gene can be analyzed in relation to available metadata [27]–[28] such as geographical location and physical and chemical environmental parameters. Although the dataset is continuing to grow, this study analyzes the first one-third of the data: the <0.8-µm size fractions from 44 marine-surface stations of a transect of the Northern Atlantic through the Gulf of Mexico and into the equatorial Pacific. In this study, we analyze the environmental diversity and biogeography of the three functionally distinct groups of LH genes within the available GOS dataset and define the environmental parameters at which different photosynthetic strategies are successful.

## Results

**Figure 1:**
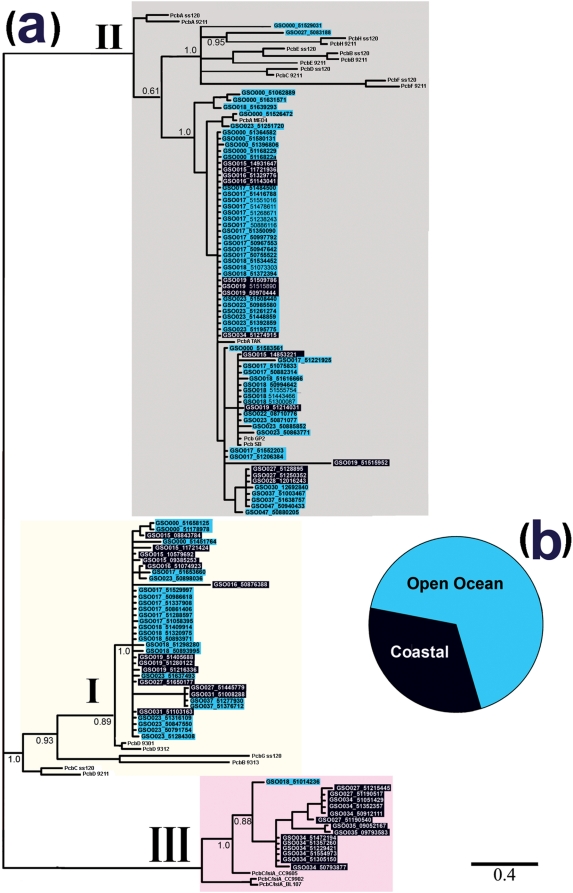
Phylogenetic analysis of the *pcb/isiA* light-harvesting gene family. A maximum-likelihood phylogenetic tree of the C-terminal region of Pcb/IsiA LH peptides (a). Pcb and IsiA proteins (sequence details see Table 1) from the sequenced representatives of *Prochlorococcus* and *Synechococcus* in the NCBI database are included as references of phylogenetic classification. The tree was rooted from the middle point. Shading indicates the environmental location of recovered sequences (coastal, dark blue; open ocean, light blue). Three phylogenetic groups are resolved, see text for details (I, gray; II, yellow; III, pink). The bar corresponds to the average substitutions per site. Bootstrapping support numbers are shown. The pie chart (b) represents the metagenomic profile of LH genes identified at open-ocean or coastal locations. Referred sequences (unshaded): PcbA_ss120, PcbA of *Prochlorococcus* sp. CCMP1375 (SS120) (NP_875175); PcbB ss120, NP_875561; PcbC_ss120, NP_875277; PcbD_ss120, NP_875559; PcbE_ss120, NP_875841; PcbF_ss120, NP_875679; PcbG_ss120, NP_875284; PcbH_ss120, NP_875566. PcbA_9211, PcbA of *Prochlorococcus* sp. MIT9211 (ZP_01005558); PcbB_9211, ZP_01005122; PcbC_9211, ZP_01005122; PcbD_9211, ZP_01005331; PcbE_9211, ZP_01004848; PcbF_9211, ZP_01004824; PcbH_9211, ZP_01005119. PcbA_MED4, PcbA of *Prochlorococcus sp* CCMP1986 (MED4) (NP_892745); PcbA_TAK, PabA of *Prochlorococcus sp* TAK9803 (AAK69281); Pcb_GB2, Pcb of *Prochlorococcus* sp. GP2 (AAK69280); Pcb_SB, Pcb of *Prochlorococcus* sp. SB (AAK69279); PcbC/IsiA_9301, PcbD of *Prochlorococcus* sp. MIT9301 (YP_001091596); PcbC/IsiA_9312, PcbD of *Prochlorococcus* sp. MIT9312 (ABB50330); PcbB_9313, PcbB of *Prochlorococcus* sp. MIT9313 (NP_894329). PcbC/IsiA_CC9605, PcbD of *Synechococcus* sp. CC9605 (YP_381894); PcbC/IsiA_CC9902, PcbD of *Synechococcus* sp. CC9902 (YP_377013); PcbC/IsiA_BL107, PcbD of *Synechococcus* sp. BL107 (ZP_01468016).

The environmental distribution of LH genes associated with different photosynthetic strategies was determined by phylogenetic analysis of all prokaryotic LH genes from marine stations of the GOS database [25] (Table 1). Of the 44 GOS stations, 19 had no hits for any prokaryotic LH peptides; these stations, representing only 14% of the sequenced GOS data, were either in coastal or temperate regions (N.E. Atlantic) and were dominated by eukaryotic cells >0.8 µm in size, or were from sites in the equatorial Pacific where the current sequenced metagenome size is very small [25]. At an *e*-value of −10, 368 unique positive hits were recovered for peptides of the Pcb or IsiA-like LH-types and 221 for those of the *Synechococcus* PBS LH-type. This study therefore identified 589 prokaryotic LH genes within the GOS dataset. Figure 1a shows the results of phylogenetic analysis of the prokaryotic chlorophyll-binding LH peptides (Pcb and IsiA-like, also referred to as accessory chlorophyll-binding proteins, CBPs [20]) in the GOS and NCBI databases (see Methods). The overall distribution of Pcb and IsiA-like peptides can be categorized into three distinct groups that reflect the phylogenetic distribution of these genes from cultured representatives [6], [19], [20], [29]. Group I comprises a group of Pcbs from *Prochlorococcus* that, owing to the results of laboratory culture experiments, are thought to act as LH antennae for the photosynthetic reaction center PSI [6], [14]–[17]. Group II comprises *Prochlorococcus* Pcbs that, from laboratory studies, are thought to act as antennae for PSII [15]. Group III is phylogenetically similar to IsiA-like peptides of marine *Synechococcus* [6], [29].

**Table 1:** The GOS station locations and environmental genomes.

Sample Dataset	Geographic Location	Genome Size MB	Pcb	PBS (only *Synechococcus*)	IsiA-like
**GS000**	Sargasso Sea	**1106**	**40**	**44**	**1**
**GS002**	North American East Coast	**150.2**		**2**	
**GS003**	North American East Coast	**108.4**			
**GS004**	North American East Coast	**92.8**		**1**	
**GS005**	North American East Coast	**107.1**			
**GS006**	North American East Coast	**104.8**			
**GS007**	North American East Coast	**89.7**			
**GS008**	North American East Coast	**160.6**			
**GS009**	North American East Coast	**98.6**			
**GS010**	North American East Coast	**96.2**			
**GS011**	North American East Coast	**155.6**			
**GS013**	North American East Coast	**173.8**			
**GS014**	North American East Coast	**163**		**5**	
**GS015**	Caribbean Sea	**160.9**	**24**	**5**	
**GS016**	Caribbean Sea	**160.3**	**8**	**1**	
**GS017**	Caribbean Sea	**455.7**	**52**	**2**	
**GS018**	Caribbean Sea	**253.6**	**43**	**2**	**3**
**GS019**	Caribbean Sea	**238.1**	**35**		
**GS021**	Eastern Tropical Pacific	**232.7**	**1**	**5**	**2**
**GS022**	Eastern Tropical Pacific	**213.5**	**6**	**5**	**3**
**GS023**	Eastern Tropical Pacific	**234.8**	**41**		
**GS026**	Galapagos Islands	**178.9**	**19**		
**GS027**	Galapagos Islands	**388.3**	**14**	**40**	**4**
**GS028**	Galapagos Islands	**333.4**	**3**	**5**	
**GS029**	Galapagos Islands	**233.1**	**2**	**3**	**1**
**GS030**	Galapagos Islands	**635.6**	**1**	**8**	**3**
**GS031**	Galapagos Islands	**758.5**	**8**	**2**	**2**
**GS034**	Galapagos Islands	**223.6**	**5**	**66**	**25**
**GS035**	Galapagos Islands	**247**	**3**	**4**	**3**
**GS036**	Galapagos Islands	**138**	**2**	**3**	
**GS037**	Eastern Tropical Pacific	**80.2**	**6**	**8**	
**GS038**	Tropical South Pacific	**0.9**			
**GS039**	Tropical South Pacific	**0.9**			
**GS040**	Tropical South Pacific	**0.9**		**1**	
**GS041**	Tropical South Pacific	**0.8**			
**GS042**	Tropical South Pacific	**0.9**			
**GS043**	Tropical South Pacific	**0.9**			
**GS044**	Tropical South Pacific	**0.8**			
**GS045**	Tropical South Pacific	**0.9**			
**GS047**	Tropical South Pacific	**80.3**	**5**	**2**	**2**
**GS048**	Polynesia Archipelagos	**1.2**			
**GS049**	Polynesia Archipelagos	**1.2**			
**GS050**	Polynesia Archipelagos	**1.2**			
**GS051**	Polynesia Archipelagos	**163.6**		**7**	**1**

The total numbers of unique genes of each defined LH gene-type identified at each station are shown. Only samples in the size fraction <0.8 µm and from surface (5-m depth) marine stations were used in this analysis; non-marine (such as a hypersaline lagoon) stations were not used. Stations where no LH genes were found are either in the NE Atlantic, and so assumed to be dominated by large eukaryotic phytoplankton species >0.8 µm, or from stations in the equatorial and south Pacific where the size of the sequenced environmental genome is low.

**Figure 2:**
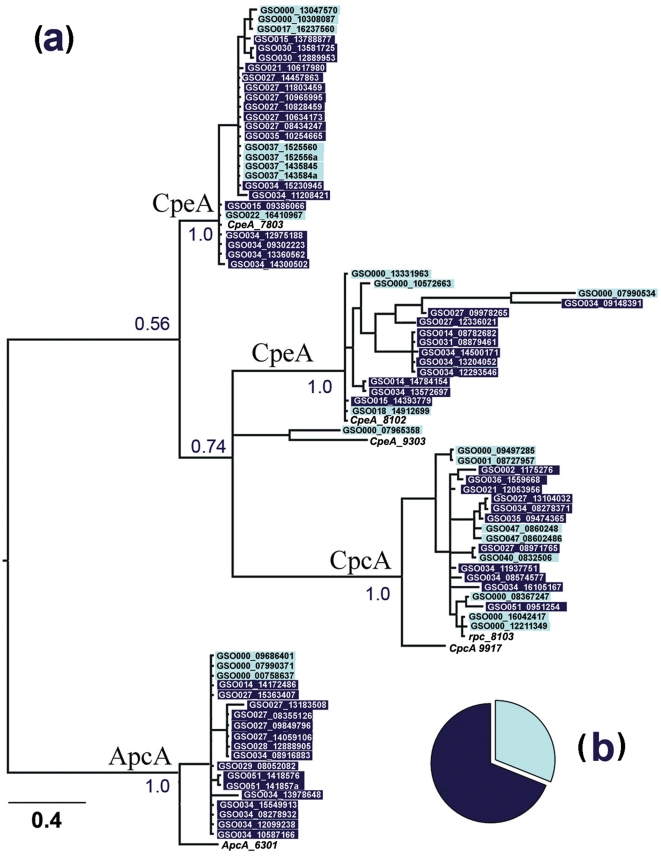
Phylogenetic analysis of the PBS light-harvesting gene family. A maximum-likelihood phylogenetic tree of the PBS alpha subunit N-terminal amino-acid sequences greater than 80 amino acids in length. (a) Phylogenetic analysis of subunit peptides (CpcA, CpeA and ApcA) obtained at the GOS stations. Shading indicates the environmental location of recovered sequences as coastal (dark blue) or open-ocean (light blue). Only one *Prochlorococcus cpeA* gene (associated with CpeA_9303) is found in the GOS dataset. The tree was rooted from the middle point. Four groups are resolved. The bar corresponds to the average substitution per site. Bootstrapping support numbers are shown. The pie chart (b) represents the metagenomic profile of LH genes identified at open-ocean or coastal locations. Referred sequences (unshaded): CpeA_7803, C-phycoerythrin class I alpha chain of *Synechococcus sp*. WH7803 (YP_001224209); CpeA 8102, C-phycoerythrin class II alpha chain of *Synechococcus sp.* WH8102 (NP_898100); CpeA_9303, Phycoerythrin alpha chain of *Prochlorococcus marinus str.* MIT9303 (YP_001018237); rpc_8103, R-phycocyanin alpha chain of *Synechococcus sp.* WH8103 (P11394); CpcA_9917, Phycocyanin alpha chain of *Synechococcus sp.* RS9917 (ZP_01080760); ApcA_6301, allophycocyanin alpha chain of *Synechococcus elongatus* PCC 6301 (YP_171896).

An important advantage of the GOS dataset is that genomic data can be analyzed in relation to the location at which the samples were obtained. Figure 1b shows that there is a greater diversity of unique genes in the *pcb/isiA*-like family at open-ocean stations compared with coastal stations. Phylogenetic studies on the PBS genes (Fig. 2 and Fig. S2) revealed similar phylogenetic relationships between environmental and cultured representatives of these genes, and shows that there is a greater diversity of PBS genes in coastal stations than open-ocean stations. This relationship was used to identify the *cpe* genes that are phylogenetically related to *cpe* genes of *Prochlorococcus* strains (Fig 2a and Fig S2 group II); as these genes are not thought to be involved in light-harvesting [13], [16] they have been omitted from further analysis.

**Figure 3:**
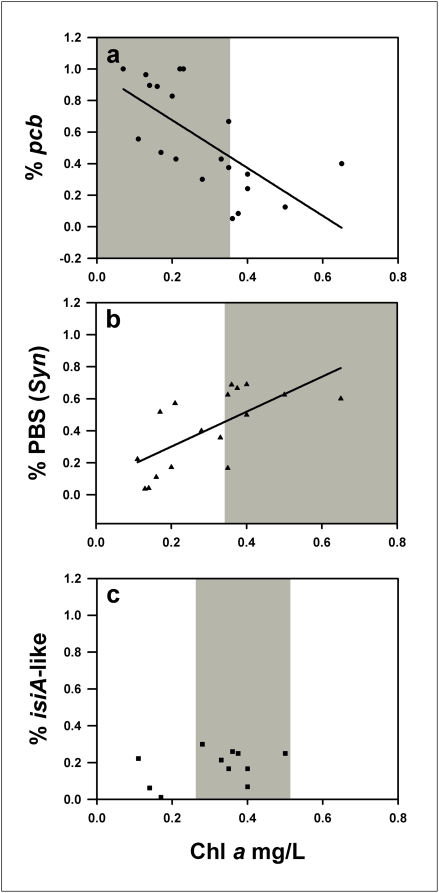
Light-harvesting gene budgets for each GOS station. Correlations of the relative diversity (at the protein level) of (a) *pcb*, (b) PBS from *Synechococcus* and (c) *isiA*-like genes recovered from the GOS stations. *pcb* genetic diversity is negatively correlated with chlorophyll concentration (n = 20 *r* ^2^ = −0.671 p<0.0005), whereas PBS genetic diversity is positively correlated with chlorophyll concentration (n = 17 *r* ^2^ = 0.669 p<0.005). *isiA*-like genetic diversity shows little correlation with chlorophyll concentration, but the *isiA*-like genes in the GOS transect are clustered at locations with surface chlorophyll concentrations of 0.26–0.51 mg/L Chl *a* (shaded areas) that separate *pcb*-dominated regions (<0.35 mg/L Chl *a*) from PBS-dominated regions (>0.35 mg/L Chl *a*).

A prokaryotic LH gene budget has been calculated by determining the fraction of the total number of functional LH genes at each GOS station that represent *pcb*, PBS or *isiA-like* LH-types. These budgets have been plotted against surface chlorophyll concentrations measured from satellite images taken at the time of sampling (Fig. 3). Chlorophyll concentration is used as a first-order indicator of phytoplankton gross biomass and can indicate that macronutrients were present in the environment; production of approximately 1 µg/L Chl *a* requires 1 µmol/L of available nitrate [30]. These plots of chlorophyll concentration and LH gene budget (Fig. 3) demonstrate that the environment selects for different photosynthetic strategies.

**Figure 4:**
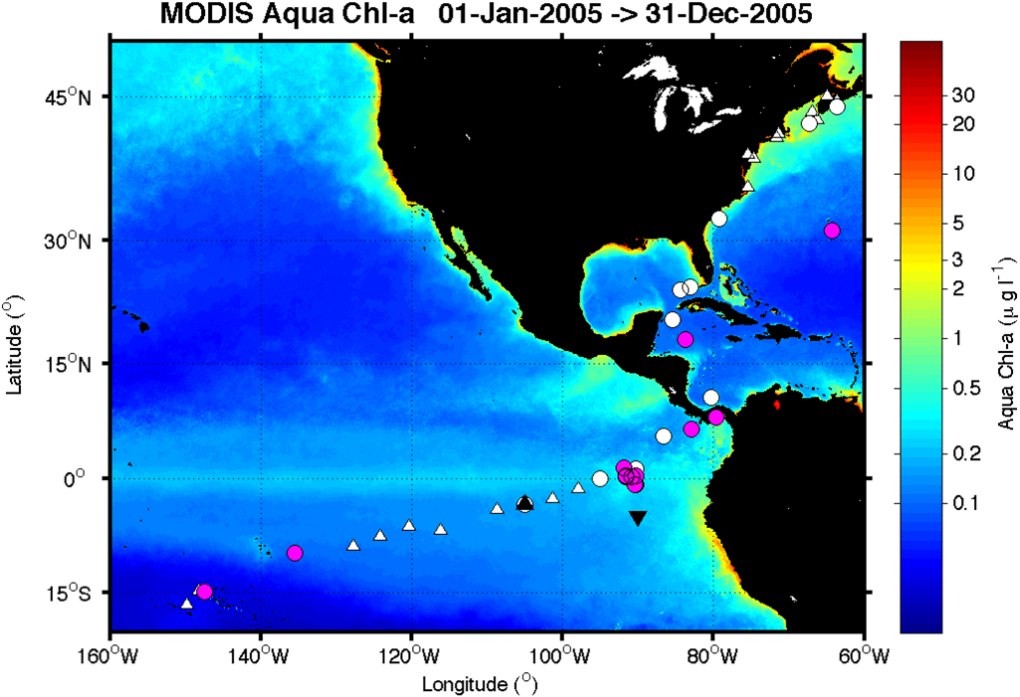
Biogeography of *isiA*-like genes. Composite of annual average surface Chl *a* concentrations (mg/L) for the global region including the GOS stations. The North and South Pacific gyres are dominated by *Prochlorococcus* and characterized by low Chl *a* concentrations. The iron-limited HNLC region dominated by *Synechococcus* extends from the coast of South America into the eastern equatorial Pacific and includes the sites of the IronExI and II experiments [32] (black arrows). White triangles indicate stations with no LH genes, white circles are GOS stations with no *isiA-like* genes, and pink circles, clustered around the Galapagos Islands, indicate the GOS stations from which at least one *isiA*-like gene was recovered.

The *pcb*-type genes show greatest genetic diversity in low-macronutrient surface waters where chlorophyll concentrations are low (<0.35 mg/L Chl *a*) (Fig. 3a). The PBS genes show greatest genetic diversity in surface waters with higher chlorophyll concentrations and increased macronutrient availability (>0.35 mg/L Chl *a*); only genes of the PBS-type were present at GOS stations with >0.7 mg/L Chl *a*, these are omitted for clarity (Fig 3b). The extent of genetic diversity within the *isiA*-like gene group shows no clear correlation with surface chlorophyll concentration (Fig. 3c); however, the distribution of this gene occupies the specific niche between environments that select for the Pcb- and PBS-type LH systems (0.26–0.51 mg/L Chl *a*). The biogeographic ranges of these LH strategies based on chlorophyll concentrations are shown in Fig. 4. The *pcb*-type LH strategy is dominant in the oligotrophic open ocean, where *Prochlorococcus* is the numerically dominant marine phytoplankton [2], [3], [8]. The PBS-type strategy is favored on the edge of the ocean gyres, in nutrient-upwelling zones and in some coastal environments, showing a good correlation with the known geographic dominance of *Synechococcus* [3], [4], [31]. Interestingly, the specific biogeography of the *isiA*-like strategy in the GOS database is in the vicinity of the Galapagos Islands, in close proximity to the sites of the classic iron-enrichment experiments IronExI and IronExII, which demonstrated that iron is the primary limiting trace element in this region [32]. The equatorial Pacific is the only prokaryotic-dominated high-nutrient low-chlorophyll (HNLC) marine ecosystem and is the only location in the GOS database at which the *isiA-like* gene is found with high genetic diversity.

## Discussion

The phylogenetic analysis of the *pcb/isiA* gene family from the GOS dataset (Fig. 1 and Fig. S1) resolves groups of functionally distinct genes similar to those recovered from analysis of genes in culture collections and environmental studies of the phylogeny of *pcb* genes [6]. This shows there is good coverage of the genetic capacity of the *pcb/isiA* gene family in current culture collections. The greater genetic diversity of the *pcb/isiA* gene family at open-ocean stations and of the PBS genes at coastal stations (Fig 1b and 2b) reflects the known global environmental distribution of *Prochlorococcus* and *Synechococcus* cells [2]–[5], and suggests that a high genetic diversity of LH functional genes reflects positive selection in a marine environment [33], [34].

*Prochlorococcus* species have been separated into two main ecotypes that are adapted to high-light (HL) or low-light (LL) conditions, with considerable further niche adaptation within these groupings [7], [14]–[16]. The PSII-type *pcb* genes are the most diverse group at all GOS sampling stations (Fig. 1a). This probably reflects the low chlorophyll content of PSII core dimers compared with PSI core trimers [15], and the resulting need for PSII to be associated with an additional LH system to increase the functional cross-section of PSII. The PSI-type Pcbs are also consistently present in surface waters throughout the GOS sampling regions, although with lower diversity (Fig. 1b). This finding is consistent with those of Kettler et al (2007), who demonstrated that many HL ecotypes of *Prochlorococcus* contain both PSI- and PSII-associated Pcbs [35]. This observation suggests that light intensity is not the main ecological selection pressure on *Prochlorococcus* photosynthetic strategy and that nutrient availability may be a more important factor in determining *Prochlorococcus* ecotype distribution [7]. Some extant representatives of *Prochlorococcus* have been shown to contain genes encoding the protein phycoerythrin (*cpeB* and *cpeA*) [13], [35], [36]. A total of 98 *Prochlorococcus cpe* genes (alpha and beta subunit incomplete sequences) were recovered from the GOS database (Fig. 2 and Fig S2), the majority of which were *cpeB*; however, the functional relevance of PBS genes in surface populations of *Prochlorococcus* is unlikely to involve light-harvesting [13], [33], so these genes were omitted from further analysis in this study.

The strong correlations between greater genetic diversity in a group of genes and surface chlorophyll concentration (Fig. 3) reflect the positive selection for LH gene-types in a particular environment [9], [31], [33], [34], [37]. In addition, these correlations are substantiated by the known energetic and functional characteristics of each LH-type. Pcb genes are dominant in low-macronutrient waters (<0.35 mg/L *Chl* a), which reflects the lower macronutrient input required for the cell to synthesize a functional *pcb*-type LH system compared with the PBS-type LH system [9], thereby making this photosynthetic strategy favoured in this environment [8], [9], [37]. At higher macronutrient concentrations (>0.35 mg/L Chl a), PBS production is energetically favoured and the PBS-type system has an advantage over Pcbs by preferentially absorbing in the range 550–650 nm, where chlorophylls cannot absorb and that are predominant in waters sustaining a high biomass [35]. At Chl *a* concentrations >0.7 mg/L, nutrient concentrations are sufficiently high to sustain large (>0.8 µm) eukaryotic phytoplankton cells that use other LH complexes [21].

The *isiA*-like LH-type is found specifically at the interface of two geographically defined regions dominated by *pcb*-type or PBS-type LH systems. Here, the environment selects for a unique photosynthetic LH strategy in which a *Synechococcus* cell incorporates both *Prochlorococcus* (Pcb type) and *Synechococcus* (PBS-type) LH antennae systems; the resulting “chimeric” cell can use each type of photosynthetic strategy and acclimate according to environmental conditions, thereby conferring a specific selective advantage and indicating that there is an environmental selection of photosynthetic strategy [31]. The observation that *isiA*-like genes are present in this region confirms that IsiA in the environment can alter the photosynthetic strategy of a cell and confer an advantage over cells with a PBS-only LH system [38]–[40]. The molecular function of the IsiA protein has been shown to be an antennae for PSI reaction centers [39], [40], increasing the functional absorption cross-sectional area by 72% and enabling iron-limited cells to reduce the ratio of PSI:PSII such that the number of PSI centers is reduced [41]. As every functional PSII contains 3 iron atoms, compared with 12 in every functional PSI, this represents a significant reduction in iron quota per cell [39]. IsiA has been used to explain the specific photophysiology of phytoplankton communities in the equatorial iron-limited surface waters and recalculate global oceanic productivity [38]. Although the biogeography of *isiA*-like genes is consistent with this description, our current understanding of the function of the IsiA protein as a coupled antenna system for PSI is at odds with this interpretation.

Of the 11 sequenced marine *Synechococcus* species, 4 have been shown to contain the *isiA*-like gene [6], [31]. Consistent with the biogeography of *isiA*-like genes outlined in this report, three of these extant marine *Synechococcus* species that contain *isiA* have been isolated from marine environments that are potentially iron-limited, including the Californian coastal upwelling zone [42] (*Synechococcus* sp CC9311, CC9605 and CC9902). *isiA* has also been reported in *Synechococcus* sp BL107, a strain isolated from ∼100-m depth in the Mediterranean; it is unlikely that surface waters of the Mediterranean are iron-limited, but there is some evidence of sub-surface iron-limitation in stratified waters, although further study is required [43].

Another marine oxyphotobacteria in which *isiA* has been found is the diazatroph *Trichodesmium* [44], [45]. *Trichodesmium* forms large colonies (>0.8 µm) and so would not be included in the current GOS dataset; however, it is widespread in many tropical and sub-tropical open-ocean gyres, where it has a key role in driving new production [44]. The distribution of *isiA*-containing *Trichodesmium* is at odds with the biogeography of the gene indicated from the GOS dataset. However, the iron requirements of *Trichodesmium* are considerably greater than those of other marine *Synechococcus* because nitrogen fixation is a major sink for iron (photosynthetic electron transfer requires 23–24 iron atoms, nitrogen fixation requires an additional 19 iron atoms) [45]. *Trichodesmium* is therefore iron-limited at greater iron concentrations than other non-diazatrophic oxyphotobacteria. The use of IsiA as an iron-efficient LH photosynthetic strategy may allow *Trichodesmium* to fix nitrogen and drive new production in many open-ocean environments.

Considering the known function of the IsiA protein, the highly restricted biogeography of *isiA* across the currently available GOS stations and the native location of sequenced marine *Synechococcus* species containing the *isiA* gene, we propose that the presence of *isiA* in the marine environment can be used as a natural biomarker of iron-limitation in prokaryotic communities [46]. This paper describes natural environmental gradients of different photosynthetic strategies in marine oxyphotobacteria on oceanic basin scales, and describes an evolutionary gradient of photosynthetic strategy from an ancestral LH system (PBS) [19], [47] that required high nutrient inputs and available iron, to a strategy that evolved to exploit increasingly iron-limited ocean environments (IsiA). Cells that permanently use this latter strategy (Pcb) could exploit the vast macronutrient-limited open-ocean gyres. Having exploited these environmental niches, photosynthetic species (using Pcbs) have become the most abundant photosynthetic species on the planet, with a pivotal role in providing energy for the marine environment.

## Materials and Methods

### Searching for LH genes in GOS

All available GOS protein sequences studied were obtained from the CAMERA database [24], [25] and were valid at the time of submission. The IsiA/Pcbs dataset was obtained by BLAST analysis of the metagenomic open-reading frame (ORF) peptide database in CAMERA (http://camera.calit2.net/) using seven selected IsiA/Pcb sequences, including Pcbs of *Prochlorococcus* sp CCMP1986 (NP_892745), *Synechococcus* sp CC9605 (YP_381894), *Prochlorococcus* sp CCMP1375 (NP_875175), *Acaryochloris marina* (AAS76629), *Acaryochloris marina* (AAS76628), and IsiA's of *Synechocystis* sp PCC6803 (NP_441268) and *Synechococcus* sp PCC7002 (P31157), with a lower cut-off (1Ex = −10); any sequences that, by compare to the sequenced NCBI data, were shown not to be *isiA/pcbs* were manually removed. The length of available GOS IsiA/Pcbs is between 50 and 352 amino acid residues. The PBS dataset was obtained by BLAST analysis of eleven sequences to the metagenomic ORF peptide database in CAMERA, including the R-phycocyanin alpha chains of *Synechococcus* sp WH8103 (P11394), C-phycoerythrin class II alpha and beta chains of *Synechococcus* sp WH8102 (NP_898100 and NP_898113), phycocyanin alpha and beta chains of *Synechococcus* sp RS9917 (ZP_01080760 and ZP_01079824), C-phycoerythrin class I alpha and beta chains of *Synechococcus* sp WH7803 (YP_001224209 and YP_001224208), phycoerythrin alpha chain of *Prochlorococcus marinus* str MIT9303 (YP_001018237), phycoerythrin beta chain of *Prochlorococcus marinus* str MIT9301 (YP_001090554), and allophycocyanin alpha and beta chains of *Synechococcus elongatus* PCC 6301 (YP_171896 and YP_171897). A total of 319 sequences related to the subunits of PBS were found in the available GOS database, of which 221 were shown by subsequent phylogenetic analysis to be similar to *Synechococcus* sequences. The length of those peptides ranged from 30 to 179 amino-acid residues. The current GOS dataset consists of sequences in the <0.8-µm cell size fraction; this will encompass all known examples of *Prochlorococcus* (cell size range 0.5–0.7 µm, mean 0.6 µm) [8] and a sizeable fraction of the *Synechococcus* species (cell size range 0.6–1.6 µm, mean 0.9 µm). The lack of sampling of some *Synechococcus* species is a limitation of the current dataset. In this study, we assume that the environmental conditions experienced by the sampled *Synechococcus* species are indicative of the entire *Synechococcus* community at that location.

### Sequence alignment

A preliminary sequence alignment was inferred by ClustalX Version 1.83 [48] with (*i*) gap opening penalty of 10.00, (*ii*) gap extension penalty of 0.2, (*iii*) the Gonnet series for protein weight matrix, and (*iv*) hydrophilic penalties for the following amino acids: G, P, S, N, D, Q, E, K and R. The alignments were refined manually based on structural information obtained from secondary structure analysis and also from crucial chlorophyll-binding amino-acid positions. The secondary structure predictions were obtained using the web based program TMHMM Version 2.0 [49]. The average full length of IsiA/Pcbs is about 350 amino acids and the average length of alpha subunits of PBS is about 164 amino acids. To obtain enough structure information, the shorter peptide sequences (shorter than 150 amino acids for Pcb/IsiA and shorter than 80 amino acids for PBS alpha subunits) were excluded from the phylogenetic tree estimation. The alignments were divided into two parts: one part including the sequences without the N-terminal region and the other part including the sequences without the C-terminal region. The known LH genes from genomes of *Prochlorococcus* and S*ynechococcus* in the NCBI dataset were used as references for the phylogenetic classification.

### Phylogenetic analysis

Phylogenetic trees were inferred by using maximum-likelihood (ML) and Bayesian methods. The ML tree was analyzed by Phyml [50]; the WAG model [51] was also used with 100 replicates to give the bootstrap values. The Bayesian method was analyzed by MrBayes Version 3.1 [52], [53]. Eight chains were run for the Metropolis-coupled Markov chain Monte Carlo model. Each chain ran for 1,000,000 generations and started with a flat prior for all trees. We have sampled trees from the chain every 100 generations. The ‘burn-in’ period covered the first 100,000 generations. A discrete-gamma model [53] was implemented to accommodate rate variation among sites for both ML and Bayesian analyses with four different categories. Phylogenetic analysis is restricted by short-length metagenomic fragments; as a result, N-terminal region or C-terminal region phylogenetic trees were constructed using data from IsiA/Pcb (>150 amino acids) and PBS (>80 amino acids), respectively. Only 24 gene sequences were recovered for the C-terminal region of PBS genes, about 10% of total recovered PBS gene fragments, so no phylogenetic tree has been constructed for these data.

### LH gene budget

LH budgets for each GOS station were determined by analysis of the proportion of the total number of unique protein fragments (of any size) recovered from each GOS station that, through sequence alignment and phylogenetic analysis, were classed as (1) Pcb, (2) IsiA-like and (3) PBS from *Synechococcus*. The statistical significance of the correlation of the proportion of each LH group at each GOS station with surface chlorophyll concentration (downloaded from the GOS dataset) was determined by Pearson's correlation on each dataset.

### Distribution of isiA in sequenced marine Synechococcus

Eleven fully sequenced and annotated genomes of *Synechococcus* were analyzed using the IMG system (http:img.jgu.doe.gov/) that also provided information on the environmental location of each species. NCBI was used to probe for the presence or absence of *isiA* homologs within these genomes.

## Supporting Information

Figure S1 A maximum-likelihood phylogenetic tree of the N-terminals of Pcb/IsiA LH peptides. The Pcb and IsiA proteins from the sequenced representatives of *Prochlorococcus* and *Synechococcus* from the NCBI database are included as references for phylogenetic classification. The tree was rooted from the middle point. Shading indicates the environmental location of recovered sequences (coastal, dark blue; open ocean, light blue). Three phylogenetic groups are resolved (for details see Fig. 1). The bar corresponds to the average substitution per site. Bootstrapping support numbers are shown. The details of reference sequences (unshaded) are given below. Referred sequences in Figure S1 PcbA_ss120, PcbA of *Prochlorococcus* sp. CCMP1375 (SS120) (NP_875175); PcbB ss120, NP_875561; PcbC_ss120, NP_875277; PcbD_ss120, NP_875559; PcbE_ss120, NP_875841; PcbF_ss120, NP_875679; PcbG_ss120, NP_875284; PcbH_ss120, NP_875566. PcbA_9211, PcbA of *Prochlorococcus* sp. MIT9211 (ZP_01005558); PcbB_9211, ZP_01005122; PcbC_9211, ZP_01005122; PcbD_9211, ZP_01005331; PcbE_9211, ZP_01004848; PcbF_9211, ZP_01004824; PcbH_9211, ZP_01005119. PcbA_MED4, PcbA of *Prochlorococcus* sp CCMP1986 (MED4) (NP_892745); PcbA_TAK, PabA of *Prochlorococcus* sp TAK9803 (AAK69281); Pcb_GB2, Pcb of *Prochlorococcus* sp. GP2 (AAK69280); Pcb_SB, Pcb of *Prochlorococcus* sp. SB (AAK69279); PcbC/IsiA_9301, PcbD of *Prochlorococcus* sp. MIT9301 (YP_001091596); PcbC/IsiA_9312, PcbD of *Prochlorococcus* sp. MIT9312 (ABB50330); PcbB_9313, PcbB of *Prochlorococcus* sp. MIT9313 (NP_894329). PcbC/IsiA_CC9605, PcbD of *Synechococcus* sp. CC9605 (YP_381894); PcbC/IsiA_CC9902, PcbD of *Synechococcus* sp. CC9902 (YP_377013); PcbC/IsiA_BL107, PcbD of *Synechococcus* sp. BL107 (ZP_01468016).
(5.80 MB TIF)

Figure S2 Phylogenetic analysis of the PBS light-harvesting gene family. (a) A maximum-likelihood phylogenetic tree of the N-terminal amino-acid sequences of PBS beta subunit peptides greater than 80 amino acids in length (CpcB, CpeB and ApcB) obtained during the GOS expedition. Shading indicates the environmental location of recovered sequences as coastal (dark blue) or open ocean (light blue). Group I refers to PBS sequences phylogenetically similar to the references sequences from *Synechococcus spp*, whereas group II refers to PBS sequences phylogenetically to *Prochlorococcus* CpeB sequences that were omitted from further analysis. The tree was rooted from the middle point. The bar corresponds to the average substitution per site. The pie chart (b) represents the metagenomic profile of LH genes identified at open-ocean or coastal locations (excluding the metagenomic sequences similar to CpeB of *Prochlorococcus spp*). The details of reference sequences (unshaded) are given in the supplementary data. Referred sequences in Figure S2: Cpeb_9301, phycobilisome protein of *Prochlorococcus marinus* str. MIT 9301 (YP_001090554); Cpeb_8102, C-phycoerythrin class I beta chain of *Synechococcus* sp. WH 8102, NP_898108; cpeb_307, C-phycoerythrin class I beta chain of *Synechococcus* sp. RCC307, YP_001228314; cpeb_7803, C-phycoerythrin class I beta chain of *Synechococcus* sp. WH 7803, YP_001224208; cpeb_9902, C-phycoerythrin class I beta chain of *Synechococcus* sp. CC9902, YP_377904; Apcb_9605, allophycocyanin beta subunit of *Synechococcus* sp. CC9605(YP_381516); Apcb_6301, allophycocyanin beta subunit of *Synechococcus elongatus* PCC 6301, YP_171897; Cpcb_9605, phycocyanin, beta subunit of *Synechococcus* sp. CC9605, YP_380752; rpc 8102, R-phycocyanin II beta chain of *Synechococcus* sp. WH 8102, NP_898113; cpcb 9917, phycocyanin beta subunit of *Synechococcus* sp. RS9917, ZP_01079824.
(6.92 MB TIF)

## References

[1] Campbell L, Vaulot D (1993). Photosynthetic picoplankton community structure in the subtropical North Pacific Ocean near Hawaii (station ALOHA).. Deep Sea Research Part I: Oceanographic Research Papers.

[2] Goericke R, Welschmeyer NA (1993). The marine prochlorophyte *Prochlorococcus* contributes significantly to phytoplankton biomass and primary production in the Sargasso Sea.. Deep Sea Research Part I: Oceanographic Research Papers.

[3] Heywood JL, Zubkov MV, Tarran GA, Fuchs BM, Holligan PM (2006). Prokaryoplankton standing stocks in oligotrophic gyre and equatorial provinces of the Atlantic Ocean: Evaluation of inter-annual variability.. Deep Sea Research Part II: Topical Studies in Oceanography.

[4] Olson RJ, Chisholm SW, Zettler ER, Armbrust EV (1990). Pigments, size, and distribution of *Synechococcus* in the North Atlantic and Pacific Oceans.. Limnology and Oceanography.

[5] Zwirglmaier K, Jardillier L, Ostrowski M, Mazard S, Garczarek L (2008). Global phylogeography of marine *Synechococcus* and *Prochlorococcus* reveals a distinct partitioning of lineages among oceanic biomes.. Environ Microbiol.

[6] Garczarek L, Dufresne A, Rousvoal S, West NJ, Mazard S (2007). High vertical and low horizontal diversity of *Prochlorococcus* ecotypes in the Mediterranean Sea in summer.. FEMS Microbiol Ecol.

[7] Johnson ZI, Zinser ER, Coe A, McNulty NP, Woodward EMS (2006). Niche partitioning among *Prochlorococcus* ecotypes along ocean-scale environmental gradients.. Science.

[8] Partensky F, Hess WR, Vaulot D (1999). *Prochlorococcus*, a marine photosynthetic prokaryote of global significance.. Microbiol Mol Biol Rev.

[9] Ting CS, Rocap G, King J, Chisholm SW (2002). Cyanobacterial photosynthesis in the oceans: the origins and significance of divergent light-harvesting strategies.. Trends Microbiol.

[10] Partensky F, Garczarek L (2003). The photosynthetic apparatus of chlorophyll *b*- and *d*-containing oxyphotobacteria.. Advances in Photosynthesis and Respiration, 14 (Photosynthesis in Algae) Kluwer Academic Publishers.

[11] Six C, Thomas JC, Garczarek L, Ostrowski M, Dufresne A (2007). Diversity and evolution of phycobilisomes in marine *Synechococcus* spp.: a comparative genomics study.. Genome Biol.

[12] Everroad RC, Wood AM (2006). Comparative molecular evolution of newly discovered picocyanobacterial strains reveals a phylogenetically informative variable region of β-phycoerytherin.. J. Phycol.

[13] Steglich C, Frankenberg-Dinkel N, Penno S, Hess WR (2005). A green light-absorbing phycoerythrin is present in the high-light-adapted marine cyanobacterium *Prochlorococcus* sp MED4.. Environ Microbiol.

[14] Garczarek L, Hess WR, Holtzendorff J, van der Staay GWM, Parrtensky F (2000). Multiplication of antenna genes as a major adaptation to low light in a marine prokaryote.. Proc Natl Acad Sci U S A.

[15] Bibby TS, Mary I, Nield J, Partensky F, Barber J (2003). Low-light-adapted *Prochlorococcus* species possess specific antennae for each photosystem.. Nature.

[16] Hess WR, Rocap G, Ting CS, Larimer F, Stilwagen S (2001). The photosynthetic apparatus of *Prochlorococcus*: insights through comparative genomics.. Photosynth Res.

[17] Bibby TS, Nield J, Partensky F, Barber J (2001). Oxyphotobacteria - antenna ring around photosystem I.. Nature.

[18] Rocap G, Larimer FW, Lamerdin J, Malfatti S, Chain P (2003). Genome divergence in two *Prochlorococcus* ecotypes reflects oceanic niche differentiation.. Nature.

[19] Burnap RL, Troyan T, Sherman LA (1993). The highly abundant chlorophyll-protein complex of iron-deficient *Synechococcus* sp. PCC7942 (CP43') is encoded by the *isiA* gene.. Plant Physiol.

[20] Chen M, Bibby TS (2005). Photosynthetic apparatus of antenna-reaction centre supercomplexes in oxyphotobacteria: insight through significance of Pcb/IsiA proteins.. Photosynth Res.

[21] Chen M, Zhang Y, Blankenship RE (2008). Nomenclature for membrane-bound light harvesting complexes of cyanobacteria.. Photosynth Res.

[22] La Roche J, van der Staay GWM, Partensky F, Ducret A, Aebersold R (1996). Independent evolution of the prochlorophyte and green plant chlorophyll a/b light-harvesting proteins.. Proc Natl Acad Sci USA.

[23] Rusch DB, Halpern AL, Sutton G, Heidelberg KB, Williamson SJ (2007). The Sorcerer II Global Ocean Sampling expedition: Northwest Atlantic through Eastern Tropical Pacific.. PLoS Biology.

[24] Venter JC, Remington K, Heidelberg JF, Halpern AL, Rusch DB (2004). Environmental genome shotgun sequencing of the Sargasso Sea.. Science.

[25] Yooseph S, Sutton G, Rusch DB, Halpern AL, Williamson SJ (2007). The Sorcerer II Global Ocean Sampling expedition: Expanding the universe of protein families.. PLoS Biology.

[26] Seshadri R, Kravitz SA, Smarr L, Gilna P, Frazier M (2007). CAMERA: A community resource for metagenomics.. PLoS Biology.

[27] Zhang Y, Gladyshev VN (2008). Trends in selenium utilization in marine microbial world revealed through the analysis of the Global Ocean Sampling (GOS) Project.. PLoS Genetics.

[28] Jensen RP, Lauro FM (2008). An assessment of actinobacterial diversity in the marine environment.. Antonie van Leeuwenhoek.

[29] Singh AK, Sherman LA (2007). Reflections on the function of IsiA, a cyanobacterial stress inducible, Chl-binding protein.. Photosynth Res.

[30] Geider RJ, MacIntyre HL, Kana TM (1996). A dynamic regulatory model of phytoplanktonic acclimation to light, nutrients, and temperature.. Limnology and Oceanography.

[31] Palenik B, Ren Q, Dupont CL, Myers GS, Heidelberg JF (2006). Genome sequence of *Synechococcus* CC9311: Insights into adaptation to a coastal environment.. Proc Natl Acad Sci U S A.

[32] Boyd PW, Jickells T, Law CS, Blain S, Boyle EA (2007). Mesoscale iron enrichment experiments 1993–2005: Synthesis and future directions.. Science.

[33] Hess WR, Partensky F, van der Staay GWM, Garcia-Fernandez JM, Boerner T (1996). Coexistence of phycoerythrin and a chlorophyll a/b antenna in a marine prokaryote.. Proc Natl Acad Sci U S A.

[34] Martiny AC, Coleman ML, Chisholm SW (2006). Phosphate acquisition genes in *Prochlorococcus* ecotype: evidence for genome-wide adaptation.. Proc. Natl. Acad. Sci. U. S. A.

[35] Kettler GC, Martiny AC, Huang K, Zucker J, Coleman ML (2007). Patterns and Implications of Gene Gain and Loss in the Evolution of *Prochlorococcus*.. PLoS Genet.

[36] Ting CS, Rocap G, King J, Chisholm SW (2001). Phycobiliprotein genes of the marine photosynthetic prokaryote *Prochlorococcus*: evidence for rapid evolution of genetic heterogeneity.. Microbiology.

[37] Palenik B, Brahamsha B, Larimer FW, Land M, Hauser L (2003). The genome of a motile marine *Synechococcus*.. Nature.

[38] Behrenfeld MJ, Worthington K, Sherrell RM, Chavez FP, Strutton P (2006). Controls on tropical Pacific Ocean productivity revealed through nutrient stress diagnostics.. Nature.

[39] Bibby TS, Nield J, Barber J (2001). Iron deficiency induces the formation of an antenna ring around trimeric photosystem I in cyanobacteria.. Nature.

[40] Boekema EJ, Hifney A, Yakushevska AE, Piotrowski M, Keegstra W (2001). A giant chlorophyll-protein complex induced by iron deficiency in cyanobacteria.. Nature.

[41] Melkozernov AN, Bibby TS, Lin S, Barber J, Blankenshp RE (2003). Time-resolved absorption and emission show that the CP43' antenna ring of iron-stressed *Synechocystis* sp. PCC6803 is efficiently coupled to the photosystem I reaction center core.. Biochemistry.

[42] Hutchins DA, DiTullio GR, Zhang Y, Bruland KW (1998). An iron limitation mosaic in the California upwelling regime.. Limnology and Oceanography.

[43] Hopkinson BA, Katherine A, Barbeau KA (2008). Interactive influences of iron and light limitation on phytoplankton at subsurface chlorophyll maxima in the eastern North Pacific.. Limnology Oceanography.

[44] Shi T, Sun Y, Falkowski PG (2007). Effects of iron limitation on the expression of metabolic genes in the marine cyanobacterium *Trichodesmium erythraeum* IMS101.. Environ Microbiol.

[45] Davis CS, McGillicuddy DJ (2006). Transatlantic abundance of the N2-fixing colonial cyanobacterium *Trichodesmium*.. Science.

[46] Geiss U, Vinnemeier J, Kunert A, Lindner I, Gemmer B (2001). Detection of the *isiA* gene across cyanobacterial strains: Potential for probing iron deficiency.. App Environ Microbiol.

[47] Zhang Y, Chen M, Zhu BB, Jermiin LS, Larkum AWD (2007). Evolution of the inner light harvesting antenna protein family of cyanobacteria, algae, and plants.. J Mol Evol.

[48] Thompson JD, Gibson TJ, Plewniak F, Jeanmouqin F, Higgins DG (1997). The Clustal_X windows interface: flexible strategies for multiple sequence alignment aided by quality analysis tools.. Nucleic Acids Res.

[49] Krogh A, Larsson B, von Heijne G, Sonnhammer EL (2001). Predicting transmembrane protein topology with a hidden Markov model: application to complete genomes.. J Mol Biol.

[50] Guindon S, Gascuel O (2003). A Simple, fast, and accurate algorithm to estimate large phylogenies by maximum likelihood.. Systematic Biol.

[51] Whelan S, Goldman N (2001). A general empirical model of protein evolution derived from multiple protein families using a maximum-likelihood approach.. Mol Biol Evol.

[52] Huelsenbeck JP, Ronquist F (2001). MRBAYES: Bayesian inference of phylogenetic trees.. Bioinformatics.

[53] Yang Z (1994). Maximum likelihood phylogenetic estimation from DNA sequences with variable rates over sites: Approximate methods.. J Mol Evol.

