# Supplementary figures and images for: Biogeography of Photosynthetic Light-Harvesting Genes in Marine Phytoplankton
Source: PLoS One. 2009 Feb 25;4(2):e4601. doi: 10.1371/journal.pone.0004601 (PMC2644788; doi:10.1371/journal.pone.0004601)

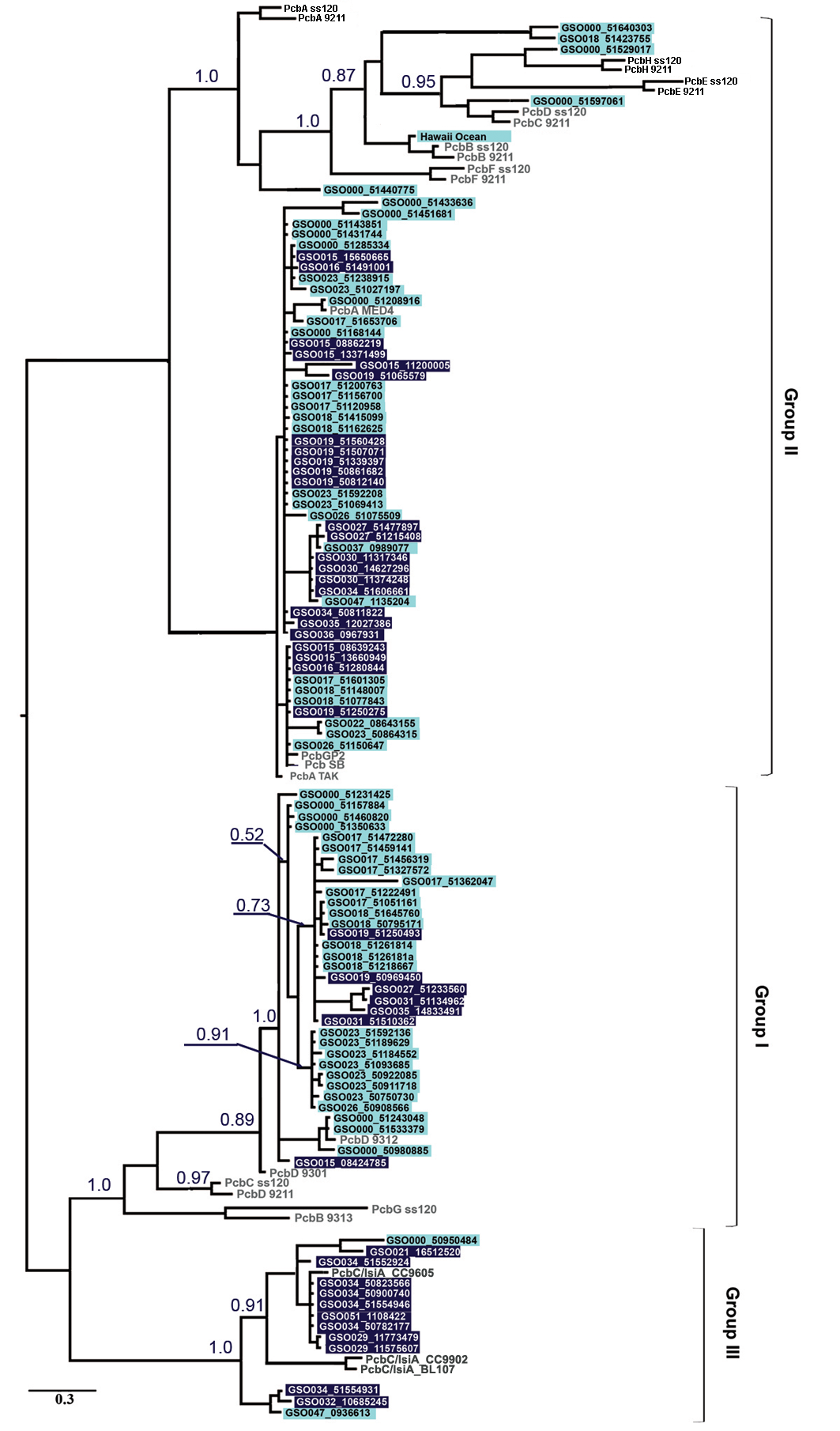

Supplement: Figure S1 — A maximum-likelihood phylogenetic tree of the N-terminals of Pcb/IsiA LH peptides. The Pcb and IsiA proteins from the sequenced representatives of Prochlorococcus and Synechococcus from the NCBI database are included as references for phylogenetic classification. The tree was rooted from the middle point. Shading indicates the environmental location of recovered sequences (coastal, dark blue; open ocean, light blue). Three phylogenetic groups are resolved (for details see Fig. 1). The bar corresponds to the average substitution per site. Bootstrapping support numbers are shown. The details of reference sequences (unshaded) are given below. Referred sequences in Figure S1 PcbA_ss120, PcbA of Prochlorococcus sp. CCMP1375 (SS120) (NP_875175); PcbB ss120, NP_875561; PcbC_ss120, NP_875277; PcbD_ss120, NP_875559; PcbE_ss120, NP_875841; PcbF_ss120, NP_875679; PcbG_ss120, NP_875284; PcbH_ss120, NP_875566. PcbA_9211, PcbA of Prochlorococcus sp. MIT9211 (ZP_01005558); PcbB_9211, ZP_01005122; PcbC_9211, ZP_01005122; PcbD_9211, ZP_01005331; PcbE_9211, ZP_01004848; PcbF_9211, ZP_01004824; PcbH_9211, ZP_01005119. PcbA_MED4, PcbA of Prochlorococcus sp CCMP1986 (MED4) (NP_892745); PcbA_TAK, PabA of Prochlorococcus sp TAK9803 (AAK69281); Pcb_GB2, Pcb of Prochlorococcus sp. GP2 (AAK69280); Pcb_SB, Pcb of Prochlorococcus sp. SB (AAK69279); PcbC/IsiA_9301, PcbD of Prochlorococcus sp. MIT9301 (YP_001091596); PcbC/IsiA_9312, PcbD of Prochlorococcus sp. MIT9312 (ABB50330); PcbB_9313, PcbB of Prochlorococcus sp. MIT9313 (NP_894329). PcbC/IsiA_CC9605, PcbD of Synechococcus sp. CC9605 (YP_381894); PcbC/IsiA_CC9902, PcbD of Synechococcus sp. CC9902 (YP_377013); PcbC/IsiA_BL107, PcbD of Synechococcus sp. BL107 (ZP_01468016). (5.80 MB TIF) [file pone.0004601.s001.tif]

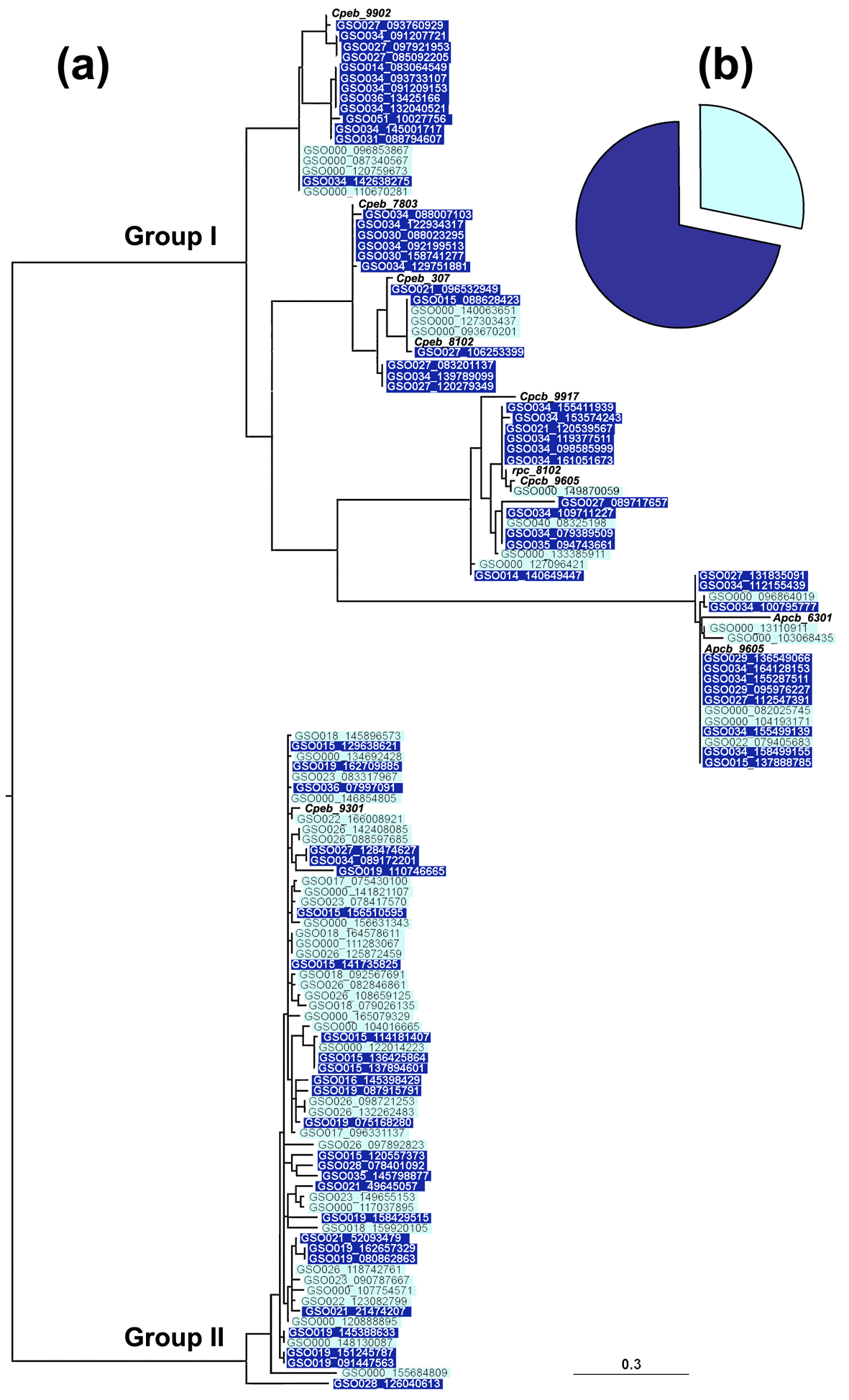

Supplement: Figure S2 — Phylogenetic analysis of the PBS light-harvesting gene family. (a) A maximum-likelihood phylogenetic tree of the N-terminal amino-acid sequences of PBS beta subunit peptides greater than 80 amino acids in length (CpcB, CpeB and ApcB) obtained during the GOS expedition. Shading indicates the environmental location of recovered sequences as coastal (dark blue) or open ocean (light blue). Group I refers to PBS sequences phylogenetically similar to the references sequences from Synechococcus spp, whereas group II refers to PBS sequences phylogenetically to Prochlorococcus CpeB sequences that were omitted from further analysis. The tree was rooted from the middle point. The bar corresponds to the average substitution per site. The pie chart (b) represents the metagenomic profile of LH genes identified at open-ocean or coastal locations (excluding the metagenomic sequences similar to CpeB of Prochlorococcus spp). The details of reference sequences (unshaded) are given in the supplementary data. Referred sequences in Figure S2: Cpeb_9301, phycobilisome protein of Prochlorococcus marinus str. MIT 9301 (YP_001090554); Cpeb_8102, C-phycoerythrin class I beta chain of Synechococcus sp. WH 8102, NP_898108; cpeb_307, C-phycoerythrin class I beta chain of Synechococcus sp. RCC307, YP_001228314; cpeb_7803, C-phycoerythrin class I beta chain of Synechococcus sp. WH 7803, YP_001224208; cpeb_9902, C-phycoerythrin class I beta chain of Synechococcus sp. CC9902, YP_377904; Apcb_9605, allophycocyanin beta subunit of Synechococcus sp. CC9605(YP_381516); Apcb_6301, allophycocyanin beta subunit of Synechococcus elongatus PCC 6301, YP_171897; Cpcb_9605, phycocyanin, beta subunit of Synechococcus sp. CC9605, YP_380752; rpc 8102, R-phycocyanin II beta chain of Synechococcus sp. WH 8102, NP_898113; cpcb 9917, phycocyanin beta subunit of Synechococcus sp. RS9917, ZP_01079824. (6.92 MB TIF) [file pone.0004601.s002.tif]
